# Case Report: Two Myxomas of Different Echodensities on Transthoracic Echocardiography in One Patient

**DOI:** 10.3389/fcvm.2021.770228

**Published:** 2022-01-20

**Authors:** Ling-Yun Kong, Xiao-Zheng Cui, Wei Xiang, Ling-Ling Chen, Li Li, Fang Liu

**Affiliations:** ^1^Department of Cardiology, School of Clinical Medicine, Beijing Tsinghua Changgung Hospital, Tsinghua University, Beijing, China; ^2^Department of Cardiovascular Surgery, School of Clinical Medicine, Beijing Tsinghua Changgung Hospital, Tsinghua University, Beijing, China; ^3^Department of Pathology, School of Clinical Medicine, Beijing Tsinghua Changgung Hospital, Tsinghua University, Beijing, China

**Keywords:** cardiac myxoma, cardio-oncology, right ventricular mass, carney complex, solid echodensity, cardiac neoplasm, pulmonary embolism

## Abstract

We report a rare case of coincidental left atrial and right ventricular myxomas manifesting as masses with different echodensities on transthoracic echocardiography. This patient had a history of left atrial myxoma, left intra-left internal carotid artery myxoma, and facial cutaneous myxoma 3 years prior to admission. A Carney complex was suspected, and the patient subsequently tested positive for *PRKAR1A* mutations. The patient was followed up regularly by a biannual echocardiography, which was free from abnormalities until the date of admission. A repeat transthoracic echocardiography revealed a massive left atrial mass of solid echodensity, and a minute hypoechoic entity in the right ventricular outflow tract. Both masses were confirmed for existence by an enhanced cardiac CT. Chest CT also revealed multiple pulmonary emboli. Successful surgical repair was performed revealing that both masses were hemorrhagic nipple-like lesions and that the pulmonary emboli were myxomatous in nature. Postoperative recovery was uneventful. Postoperative echocardiography showed a clear heart chamber, and the 1-year follow-up showed no abnormalities. Further research is needed to clarify the echocardiographic characteristics of multiple myxomas when they occurred simultaneously in different chambers.

## Introduction

Cardiac myxoma has long been recognized as the most common primary cardiac neoplasm (1). A typical cardiac myxoma is located in the left atrium, attached to the interatrial septum, and exhibits a solid echodensity on echocardiography. Herein, we present a case of recurrent myxoma and pulmonary tumor embolism in a patient with Carney complex. Echocardiography revealed left atrial and right ventricular outflow tract masses with different echodensities. Re-open chest surgery showed that both masses had a similar hemorrhagic appearance. Histopathology confirmed that both cardiac masses were myxomas, and the pulmonary emboli contained myxomatous cells.

## Case Presentation

A 52-year-old man was presented in June 2020 with a complaint of intermittent chest discomfort and back pain in a 3-week duration. His past medical history included admission at another institution for an episode of syncope 3 years prior. This was found to be associated with left atrial (LA) mass and left intra-left internal carotid artery mass that were resected and confirmed to be myxomas. He was also diagnosed with a facial cutaneous myxoma, cerebral infarction, and right renal atrophy. Screening for *PRKAR1A* mutations was positive, and a diagnosis of the Carney complex (CNC) was confirmed.

**Figure 1:**
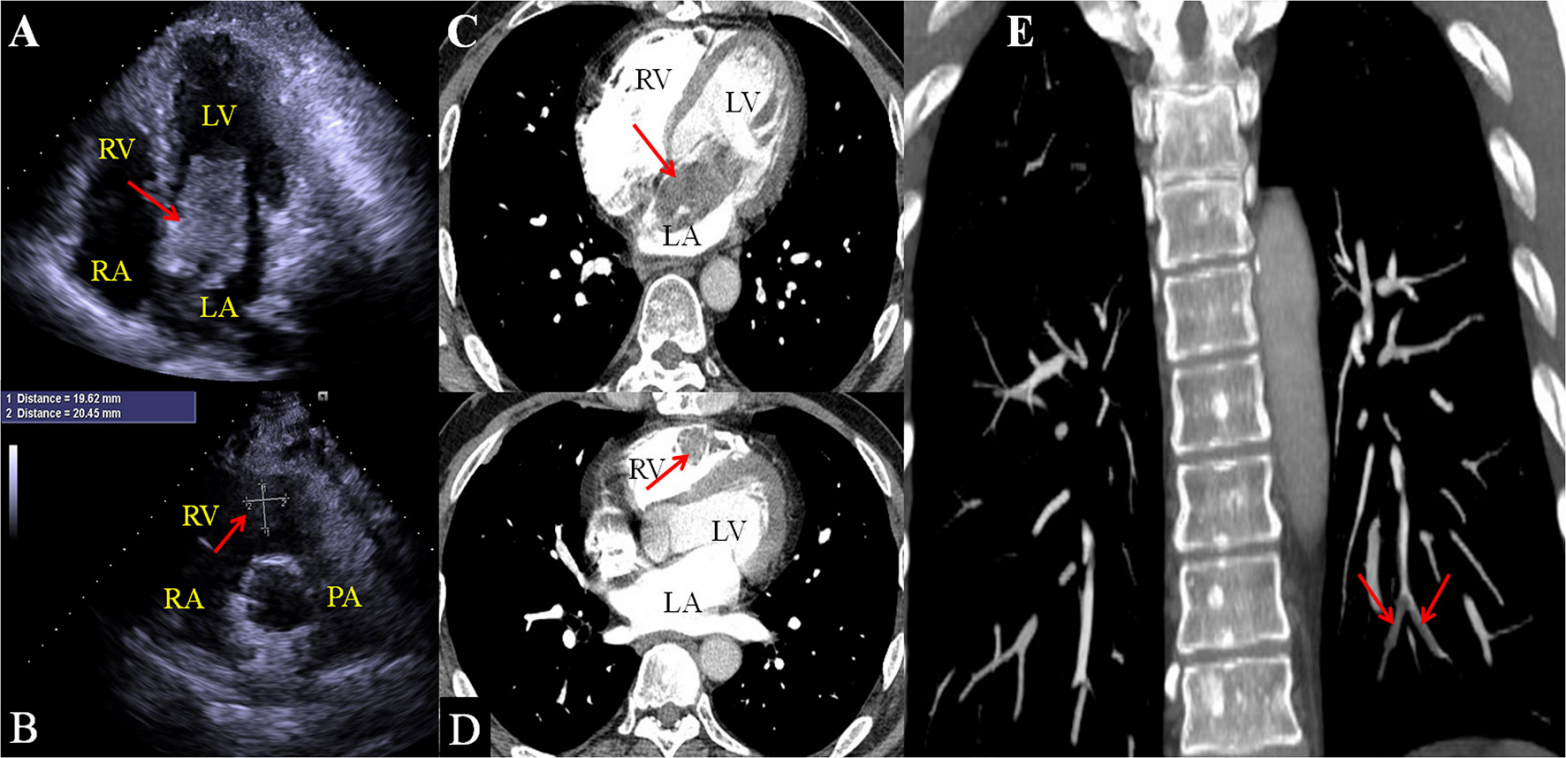
2D echocardiography shows an irregular hyper-echoic mass in the left atrium **(A)** and a hypoechoic mass in the right ventricular outflow tract **(B)**. Enhanced chest CT confirms the LA mass **(C)** and right ventricular mass **(D)**. CT Pulmonary arteriography confirms that the nodules on regular CT are pulmonary emboli [**(E)**, red arrows].

**Figure 2:**
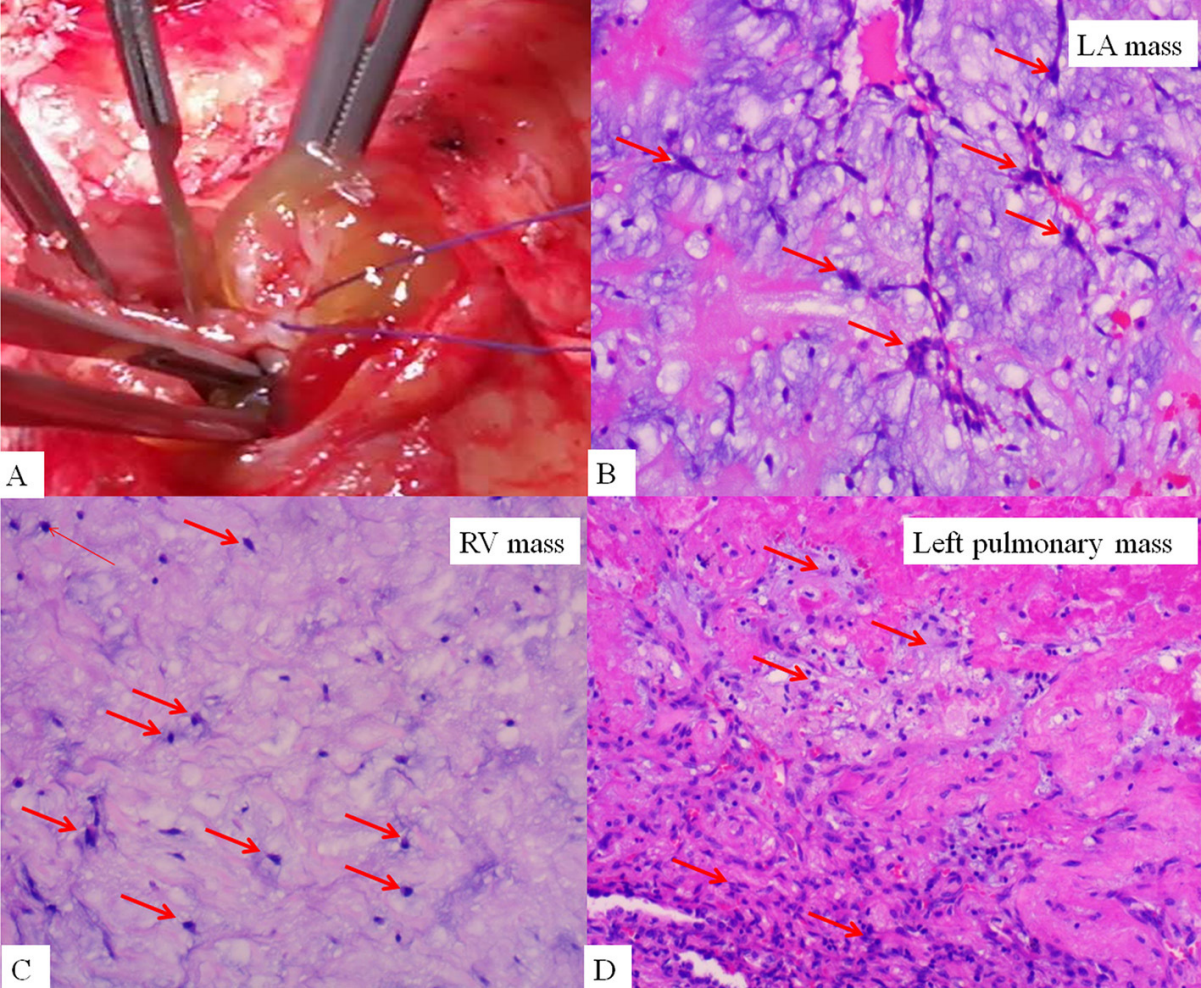
Gross finding of right ventricular mass **(A)**, which is nipple-shaped, jelly-like, and pedunculated, similar to the mass in the LA. Histopathological examination confirmed the LA mass **(B)**, right ventricular mass **(C)**, and left pulmonary mass **(D)** to be rich in myxomatous cells.

On admission, the vital signs of the patient were stable. Physical examination revealed a facial pigmentation and a scar at the right nasolabial groove, consistent with the history of excision of a facial cutaneous myxoma. No abnormalities were detected on cardiac and respiratory auscultation, and no lower limb edema was observed. Laboratory testing revealed an elevated serum creatinine (105.5 μmol/L), corresponding to an estimated glomerular filtration rate of 69.6 ml/min/1.73 m^2^. Electrocardiography showed nonspecific T wave changes. Transthoracic echocardiography revealed a large (62 mm × 28 mm), pedunculated, and highly mobile hyperechoic mass in the LA (Figure 1A; Supplementary Video 1) attached to the middle interatrial septum. The mass crossed the mitral valve into the left ventricle during diastole and returned to the LA during systole. No obstruction to the left ventricular inflow was found, and the mass appeared to have variable shapes and sizes with cardiac cycles. Additionally, another hypo-echodensity was detected, although nearly missed in the right ventricular (RV) outflow tract (Figure 1B, Supplementary Video 2). This mass was also redundant and appeared to have variable shape and size with cardiac cycles. No obstruction to the RV outflow tract was observed. Both masses in the LA and the RV were confirmed by an enhanced cardiac CT (Figures 1C,D). Quantitative analysis showed that the CT value of the LA myxoma was about 19.8 HU, close to the water's density, while the RV myxoma has a CT value of about 93.5 HU, higher than the soft tissue. However, chest CT upon admission (performed in accordance with the admission policy during COVID-19 pandemic) showed multiple nodules in the left lower pulmonary lobe, which were confirmed to be pulmonary artery emboli by a CT pulmonary angiography (Figure 1E). The patient underwent successful surgical excision of the LA mass, RV mass, and resection of the left lower lung. Both LA and RV (Figure 2A) masses were similar in appearance which appeared to be nipple-shaped and jelly-like with stalks. Pathologic investigation confirmed that tissues from the LA (Figure 2B), RV (Figure 2C), and pulmonary artery (Figure 2D) were all rich in myxomatous cells. The LA and RV myxomas had similar myxomatous component and density, and, yet, the myxoma in LA was richer in vessels and had more congestive and hemorrhagic areas, consistent with its low CT value. The patient was advised to be closely followed up with biannual echocardiography, and the 1-year follow-up was negative.

**Table:** 

2017	The patient was admitted for syncope. A left atrial mass and a left intra-left internal carotid artery mass were detected, and both were confirmed to be myxomas. Facial cutaneous myxoma, cerebral infarction, and right renal atrophy were also identified. Carney complex was considered, and the PRKAR1A mutation test was positive.
2018–2019	Normal findings on a regular biannual echocardiographic follow-up.
June 2020	The patient complained of chest discomfort and back pain. Repeat echocardiogram revealed a mass in the left atrium and another mass in the right ventricle. CT confirmed the presence of the masses and revealed multiple pulmonary nodules. Re-open heart surgery was successfully performed to remove the masses and the left lower lung lobe.
2021 (1 year after discharge)	Normal findings on a regular biannual echocardiographic follow-up.

## Discussion

We describe a case of recurrent cardiac myxomas in a patient with Carney complex, which was diagnosed at one institution but followed up at another. The initial cardiac myxoma occurred in the LA, and 3 years after the first operation, myxomas recurred in the LA and the RV outflow tract which were confirmed by histopathological examination. This case is unique in that, although the masses identified on a repeat echocardiography had a similar gross appearance and the same pathological presentations. They demonstrated different echodensities on transthoracic echocardiography, which is the most common technique for detection and follow-up of cardiac masses.

Cardiac myxoma is the most common non-cutaneous lesion in CNCs (20–40%) (2). The clinical manifestation of cardiac myxoma is heterogeneous and related to the intracardiac blood flow obstruction or embolic phenomena. It typically occurs in younger patients with atypical and multiple foci, appears to grow linearly over time (3), and tends to have a higher risk of distal embolism (4–6) and postoperative recurrence. Therefore, for patients with an initial diagnosis of myxoma, a systematic examination involving physical examination for evidence of facial pigmentation or myxoma, breast nodule, and endocrine ultrasonography or CT is necessary to reveal more information. Early surgery upon identification is suggested due to the tumor's high risk of embolization. Additionally, since the spectrum of CNCs is broad and some may develop with aging, it is advisable for the patient to be followed up closely and regularly (3).

There are numerous reports on recurrent cardiac myxomas (2, 4, 5), however, only few have discussed the echocardiographic characteristics, especially when they occur in two chambers (7). Additionally, the differential diagnosis of the right heart masses included thrombus (8), metastasis, lipoma, fibroma, rhabdomyoma, and lymphoma (9). None of these has unique imaging characteristics, and the initial judgment of echocardiographers, which, based on experience, is sometimes expected by clinicians. Although for patients with a diagnosis of CNC, recurrent cardiac masses should initially raise suspicion of myxomas, concurrent cardiac myxomas of various echodensities in different chambers at the same examination pose a challenge for clinicians to make a confident initial diagnosis. In this case, the patient was diagnosed at one institution and followed up at another, and the sonographers and clinicians may be unfamiliar with the medical history of the patient, or unclear about the clinical characteristics of CNCs. The reason for different echodensity of myxomas in the LA and in the RV might be associated with the site and orientation of the tumor cells relative to the ultrasound beam, similar to the case in the change of echogenicity of the interventricular septum in the long axis views vs. in the short axis views. The difference cannot be explained by the underlying similar histopathologic characteristics, as the hyper-echogenic myxoma in the LA was actually the one that was richer in vessels and congestive and hemorrhagic areas, and had lower density on CT in contrast with the hypo-echogenic myxoma in the RV.

This case describes the echocardiographic characteristics of a cardiac myxoma. It also provides evidence for a frustrating prognosis and invasive trend of cardiac myxoma in patients with *PRKAR1A* mutations (7). This patient suffered both systemic (renal infarction and cerebral infarction) and pulmonic embolism. Echocardiography is the key to identifying cardiac myxomas, as well as in assessing their structural and hemodynamic effect. Clinicians should be aware that intracardiac masses of different echodensities can have the same pathological origin. The established treatment was surgical excision. More experience is needed to better understand the imaging characteristics of myxomas in different cardiac chambers.

## Conclusions

Cardiac myxoma has a high risk of recurrence after surgical excision. Recurrent and multiple cardiac myxomas in different cardiac chambers may exhibit different echogenicities even in the same patient at the same examination time. Close follow-up is required in this patient group to achieve early detection and to protect patients from the complications of recurrence of myxomas.

## Data Availability Statement

The original contributions presented in the study are included in the article/Supplementary Material, further inquiries can be directed to the corresponding author/s.

## Ethics Statement

Written informed consent was obtained from the relevant individual for the publication of any potentially identifiable images or data included in this article.

## Author Contributions

L-YK contributed to the clinical design and concept. L-YK, WX, and X-ZC acquired clinical data. L-LC performed the echocardiographic examinations. LL performed pathological analyses. L-YK and FL interpreted the data and drafted and revised the manuscript. All authors critically revised the manuscript and approved the final version.

## Funding

This work was sponsored by Beijing Tsinghua Changgung Hospital Startup Foundation for Young Scientists (Grant No. 12021C1004).

## Conflict of Interest

The authors declare that the research was conducted in the absence of any commercial or financial relationships that could be construed as a potential conflict of interest.

## Publisher's Note

All claims expressed in this article are solely those of the authors and do not necessarily represent those of their affiliated organizations, or those of the publisher, the editors and the reviewers. Any product that may be evaluated in this article, or claim that may be made by its manufacturer, is not guaranteed or endorsed by the publisher.

## Supplementary Material

Supplementary Video 1 Echocardiography apical 4-chamber view shows a large (62 mm × 28 mm) and highly mobile hyperechoic mass in the left atrium.

Supplementary Video 2 Parasternal short axis view reveals a hypoechoic mass about 25 mm × 22 mm attached to the free wall of the right ventricular outflow tract.
